# Long-term outcome of endovascular repair of thoracic and abdominal aortic diseases: a retrospective cohort study of 101 patients from a tertiary care centre

**DOI:** 10.1590/1677-5449.202401472

**Published:** 2025-07-30

**Authors:** Rajesh Vijayvergiya, Basant Kumar, Lipi Uppal, Ashish Sharma, Ajay Savlania, Ankush Gupta, Harkant Singh, Manphool Singhal

**Affiliations:** 1 Post Graduate Institute of Medical Education and Research - PGIMER, Chandigarh, India.

**Keywords:** endovascular repair of abdominal aorta (EVAR), thoracic endovascular aortic repair (TEVAR), (thoraco-abdominal) endovascular aortic repair [(T)EVAR], Reparação endovascular da aorta abdominal (EVAR), reparação endovascular da aorta torácica (TEVAR), reparação endovascular da aorta (toracoabdominal) [(T)EVAR]

## Abstract

**Background:**

Thoraco-abdominal endovascular aortic repair [(T)EVAR] of thoracic (TEVAR) and abdominal aorta (EVAR) has surpassed open surgical repair for thoraco-abdominal aortic diseases.

**Objectives:**

We describe the long-term outcomes of 101 (T)EVAR patients treated over the last eleven years.

**Methods:**

A retrospective analysis of 101 consecutive (T)EVAR patients was performed. The primary endpoints were in-hospital and 30-day outcomes, while the secondary endpoints were long-term outcomes and re-intervention rates.

**Results:**

Out of 101 patients, EVAR and TEVAR were performed in 40 (39.6%) and 61 (60.3%) patients, respectively. Mean age was 58.04 ± 15.7 years. Technical success rates were 100% and 95% in the EVAR and TEVAR groups, respectively. Intraoperative endoleak was observed in 17 patients. Major perioperative complications (n=16) included retrograde aortic dissection (n=1), stent graft migration (n=2), paraparesis (n=1), device system entrapment in iliac vessels (n=1), acute renal failure (n=2), acute limb ischemia (n=3), and aorto-enteric fistula (n=2). The 30-day mortality rate was 7.9% (8 patients). Kaplan Meyer survival estimates at 1 and 5 years were 79% (95% CI 66.0-87.0, SE 0.053%) and 71% (95%CI 56.0- 81.0, SE 0.065%) for TEVAR and 84% (95% CI 67.0-92.0, SE 0.061%) and 69% (95%CI 46.0-83.0, SE 0.094%) for EVAR, respectively. Diabetes and smoking were associated with increased all-cause mortality in EVAR (p=0.018) and TEVAR (p=0.045) cases, respectively, following Cox regression analysis.

**Conclusions:**

We observed favorable short- and long-term outcomes in 101 (T)EVAR patients, proving its safety and long-term efficacy for management of thoracoabdominal aortic disease.

## Resumo

**Contexto:**

Os reparos endovasculares da aorta torácica (TEVAR) e da aorta abdominal (EVAR) superaram a cirurgia aberta no tratamento das doenças da aorta toracoabdominal.

**Objetivos:**

Descrever os desfechos a longo prazo de 101 pacientes submetidos a TEVAR e EVAR nos últimos 11 anos.

**Métodos:**

Foi realizada uma análise retrospectiva de 101 pacientes consecutivos submetidos a TEVAR/EVAR. Os desfechos primários foram os resultados intra-hospitalares e aos 30 dias, enquanto os desfechos secundários incluíram os resultados a longo prazo e as taxas de reintervenção.

**Resultados:**

Dos 101 pacientes, EVAR e TEVAR foram realizados em 40 (39,6%) e 61 (60,3%), respectivamente. A idade média foi de 58,04±15,7 anos. A taxa de sucesso técnico foi de 100% no grupo EVAR e de 95% no grupo TEVAR. Vazamento interno intraoperatório foi observado em 17 pacientes. As principais complicações perioperatórias (n=16) incluíram dissecção aórtica retrógrada (n=1), migração da endoprótese (n=2), paraparesia (n=1), aprisionamento do sistema do dispositivo nos vasos ilíacos (n=1), insuficiência renal aguda (n=2), isquemia aguda de membros (n=3) e fístula aortoentérica (n=2). A mortalidade aos 30 dias foi registrada em oito (7,9%) pacientes. As estimativas de sobrevida de Kaplan-Meier em 1 e 5 anos foram, respectivamente: 79% (IC95% 66,0-87,0; SE 0,053%) e 71% (IC95% 56,0-81,0; SE 0,065%) no grupo TEVAR; e 84% (IC95% 67,0-92,0; SE 0,061%) e 69% (IC95% 46,0-83,0; SE 0,094%) no grupo EVAR. Diabetes e tabagismo foram associados a um aumento da mortalidade por todas as causas nos grupos EVAR (p=0,018) e TEVAR (p=0,045), respectivamente, após análise de regressão de Cox.

**Conclusões:**

Observamos desfechos favoráveis a curto e longo prazo em 101 pacientes submetidos a TEVAR/EVAR, comprovando sua segurança e eficácia a longo prazo no tratamento das doenças da aorta toracoabdominal.

## INTRODUCTION

Thoraco-abdominal aortic disease is often referred to as a “silent killer” because of the relatively asymptomatic nature of the disease and its devastating outcomes.^1^ Many times, the first manifestation of an aortic aneurysm may be death or a significant complication in the form of dissection and perforation that warrants emergent repair. Surgical repair was historically the gold standard for many decades, before endovascular repair was introduced in the 1990s.^2^ Since then, endovascular procedures have surpassed the number of open surgical repairs, and 75% - 80% of patients are now being treated with (T)EVAR.^3,4^

The current literature has demonstrated excellent perioperative benefits of (T)EVAR, with lower overall mortality, avoiding the need for thoracotomy, shorter ICU stays, and lower operative blood loss.^5^ The improved safety profile, advanced stent graft technology, and growing physician expertise have improved outcomes in recent years. (T)EVAR has become the treatment of choice for patients with suitable aortic anatomy.^6^ However, there remains uncertainty over mid- and long-term outcomes, with more extensive studies using the Medicare database showing a loss of survival advantage as early as 2-3 years.^7,8^ Endovascular grafts may be at higher risk of long-term failure due to complications such as graft migration, stenosis, endoleak, progressive sac enlargement, and secondary ruptures.^6^ Re-intervention rates, primarily for endoleak, remain high in patients treated with (T)EVAR, requiring diligent follow-up.^6^

This lacuna regarding the extended follow-up of patients following (T)EVAR thus remains unaddressed due to a lack of data, especially from the Indian subcontinent, where late presentations, resource-limited settings, higher comorbidity burden, and small caliber vessels offer unique challenges for endovascular interventions. This study retrospectively examined the outcomes of over a hundred patients with endovascular aortic interventions performed in the last decade at a single tertiary care center in North India.

## MATERIALS AND METHODS

**Figure 1:**
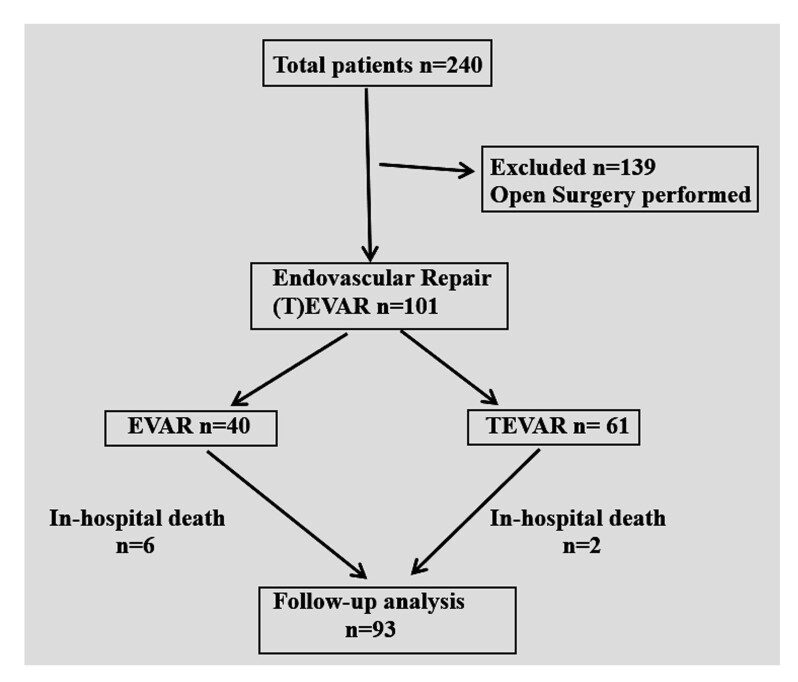
Flow chart of aortic disease patients subjected to intervention (open surgical plus endovascular) at our institute over the last 11 years.

This retrospective cohort study included 101 consecutive patients who underwent endovascular repair at our institute over eleven years from 2012 to 2023. Indications for T(EVAR) included symptomatic aortic aneurysms, acute and chronic type B dissections, pseudoaneurysms with impending ruptures, aortic-enteric fistulas, aortobronchial fistulas, and traumatic injuries. Patients with ascending aorta and abdominal aorta with visceral artery involvement who required branched or fenestrated grafts were excluded from the study. These patients were treated with open surgical repair. We had a total of 240 patients with aortic diseases over the last eleven years, 139 of whom underwent open surgical repair while the remaining 101 underwent T(EVAR) (Figure 1). Demographic and operative details, including device access site, device size, method of deployment, presence of endoleak, and in-hospital complications, were collected from patients’ medical records maintained for the individual patients. Follow-up data were collected via in-hospital medical records and patients not on follow-up were individually contacted telephonically or via personal communication.

Acute aortic dissection was defined as dissection within the last 14 days. Complicated aortic pathologies requiring urgent intervention included impending ruptures, ruptures with end-organ malperfusion, aorto-esophageal fistulas and traumatic injury. Technical success was defined as successful device deployment at the intended location without evidence of type I or type III endoleak in the final angiogram and hemodynamic stability for at least 24 hours postoperatively.^9^ Reinterventions were defined as any surgical or endovascular procedure required during follow-up for existing aortic pathology. An endoleak was defined as radiological evidence of flow outside the stent graft and was classified according to current guidelines.^9^ The study’s primary endpoints were in-hospital and 30-day outcomes, while the secondary endpoints were long-term survival and re-intervention rates. After discharge, patients were followed up at 1, 3, 6, and 12 months and annually after that. A routine computed tomography (CT) scan was performed 6-12 months after the index procedure and was repeated after that if clinically required. The study was in accordance with the Helsinki Convention and approved by the institutional ethics committee for the retrospective analysis, vide no. INT/IEC/2025/SPL-1543.

Categorical variables were expressed as numbers and percentages n (%); normally distributed continuous variables were expressed as mean and standard deviation. As part of a prespecified analysis, the individual variables found to be significant in univariate analysis were used in multivariate logistic regression analysis to determine long-term mortality and reintervention rates. The analysis was conducted on an intention-to-treat population, and a sensitivity analysis was performed to assess the robustness of the findings. All p-values were two-sided, and p values <0.05 were considered statistically significant. Overall survival was determined using Kaplan-Meier methods, while log-rank tests were used to compare survival between the two groups. All statistical analyses were conducted using IBM Statistical Product and Service Solutions (SPSS) version 26.

## RESULTS

Out of a total of 101 T(EVAR) patients, TEVAR and EVAR were performed in 61 and 40 patients, respectively. Mean patient age was 52.8 ± 15.2 years and 66 ± 12.9 years in the TEVAR and EVAR groups, respectively. Males comprised 78.2% of the study population. The baseline characteristics of the groups are illustrated in Table 1. The most common risk factor was hypertension, present in 42 (68.8%) and 29 (72.5%) patients in the TEVAR and EVAR groups, respectively. Four (6.5%) patients in the TEVAR group had undergone previous open surgical repair of the aortic arch- three had undergone Bentall’s procedure, and one had had post-traumatic aortic transection repair. Fifteen (14.8%) patients underwent emergency procedures due to acute dissection (n=3), traumatic ruptures (n=3), impending ruptures (n=4), and aortobronchial (n=3) and aorto-esophageal fistulas (n=2).

**Table 1:** Baseline characteristics of patients in thoracic (TEVAR) and abdominal (EVAR) endovascular repair groups.

**Characteristics**	**TEVAR (n=61)**	**EVAR (n=40)**	**Total (n=101)**	**p-value**
Age, in years	52.8 ±15.2	66±12.9	58.04±15.7	0.001
Male sex, *n* (%)	45 (73.0)	34 (85)	79(78.2)	0.223
Diabetes Mellitus, n (%)	9 (14.7)	8 (20)	17(16.8)	0.589
Hypertension*, n* (%)	42 (68.8)	29 (72.5)	71(70.2)	0.395
Chronic kidney disease, *n* (%)	6 (9.8)	02 (5)	08 (7.9)	0.381
Post renal transplant	01 (1.6)	02 (5)	03 (2.9)	0.333
History of smoking, *n* (%)	21 (34.4)	26 (65)	47 (46.5)	0.004
Peripheral arterial disease, *n* (%)	3 (4.9)	3 (7.5)	06 (5.9)	0.593
Coronary artery disease, *n* (%)	10 (16.3)	16 (40)	26 (25.7)	0.008
Cerebrovascular accidents	0 (0)	02 (5)	02 (1.9)	0.079
Previous aortic repair	4 (6.5)	0 (0)	04 (3.9)	0.10
Bentall procedure	03 (4.9)			
Open aortic repair for traumatic rupture	01 (1.6)			
Past/present history of tuberculosis	3 (4.9)	0 (0)	03 (2.9)	0.157
Connective tissue disorder	3 (4.9)	0 (0)	03 (2.9)	0.157
Emergency procedures, *n* (%)	13 (21.3)	2 (5)	15 (14.8)	0.038

All procedures were performed under general anesthesia by a team of interventional cardiologists, vascular surgeons, and cardiac anesthesiologists. Vascular access to the common femoral artery was obtained via cut-down in 89 (88.1%) patients and 8 (7.9%) patients required a retroperitoneal open iliac conduit to the right common iliac artery (n=7) and aortic bifurcation (n=1) due to unfavorable iliofemoral anatomy.^10^ The study used two different stent graft systems with no specific preference. In patients undergoing TEVAR, 24 (39.3%) patients were treated with the Valiant thoracic graft stent graft system (Medtronic Vascular, Santa Rosa, California), and 37 (60.6%) patients received Cook endovascular stent grafts (Cook Inc, Bloomington, IN). Among 40 patients undergoing EVAR, the Endurant stent graft system (Medtronic Vascular, Santa Rosa, California) was used in 17 (42.5%), and the rest were treated with Cook endovascular stent grafts (Cook Inc, Bloomington, IN).

The procedural characteristics are shown in Table 2. The procedural success rate in patients undergoing TEVAR was 95%, with 55 (90%) requiring single graft stents. The location of the proximal site attachment and length of the coverage was dependent on the complexity of the procedure, pre-procedure CT scan assessment, identification of the stressors placed on the device, and anticipated complications. Ishimaru’s classification of five landing zones, depending upon the distal border of each aortic arch vessel, was considered for stent deployment during TEVAR.^9^ Of 61 TEVAR patients, 33 (54%) underwent subclavian artery coverage with graft placement in zone 2. Three patients underwent zone 1 stent placement following open surgical carotid artery bypass surgery, and two patients underwent zone 0 stent placement following a total surgical debranching procedure. The remaining 23 patients had stent graft placements distal to the left subclavian artery. Major perioperative complications were observed in 9 (14.7%) patients, with 6 (9.8%) patients succumbing during the hospital stay. Life-threatening complications included retrograde aortic dissection (n=1), distal entry tear with descending thoracic aorta rupture (n=1), aorto-esophageal fistula (n=1), acute renal injury with sepsis (n=2), and device delivery system entrapment in iliac vessels requiring open surgical repair (n=1) (Table 2). Two patients underwent emergency TEVAR for ruptured thoracic pseudoaneurysm with aorto-enteric fistula. The first case required surgical re-exploration five days after TEVAR for pus debridement and the patient later succumbed to sepsis. The second patient, treated for mycotic pseudoaneurysm and aorto-esophageal fistula, presented with stent graft infection and recurrence of aorto-esophageal fistula at 12 months of follow-up and could not be saved.^11^ Three patients with thoracic pseudoaneurysm and aortobronchial fistula presented with massive hemoptysis and underwent emergency TEVAR.^12^

**Table 2:** Procedural characteristics of two groups, TEVAR and EVAR.

**Procedural characteristics**	**TEVAR (n=61)**		**EVAR (n=40)**	
Indications	Aneurysm	12 (19.6)	Aneurysm	38 (95)
	Dissection	39 (63.9)	Pseudoaneurysm	02 (05)
	Pseudoaneurysm with impending rupture	2 (3.2)		
	Aorto-esophageal fistula	2 (3.2)		
	Aortobronchial fistula	3 (4.9)		
	Traumatic	3 (4.9)		
Stent graft systems	Valiant stent graft	24 (39.3)	Endurant stent graft	17 (42.5)
	Cook Zenith stent	37 (60.6)	Cook Zenith stent graft	23 (57.5)
Procedural	Stent graft placement in zone 3	23 (37.7)	Bilateral EVAR	36 (90)
	Left subclavian artery coverage (zone 2)	33 (54.0)	EVAR and fem fem surgical bypass	4 (10)
characteristics	Preop carotid subclavian artery bypass (zone 1)	3 (4.9)	Hybrid EVAR with surgical bypass	01
	Total debranching procedure (zone 0)	2 (3.2)		
Vascular access				
Percutaneous	2 (3.2)		2 (05)	
Surgical	54 (88)		35 (87)	
Surgical with conduit	5 (8.1)		3 (7.5)	
Mean postoperative hospital stay, days (±1SD)	7.3±5.4		8.3 ± 12.6	
No. of patients required post-intervention blood transfusion	3 (4.9)		2 (05)	

In the EVAR group, thirty-six patients had bilateral EVAR, while 4 had uni-iliac EVAR with femoral-femoral bypass. One post-renal transplant patient had an open surgical bypass graft to the superior mesenteric artery before EVAR.^13^ The success rate of EVAR was 100%, with two patients succumbing in the postoperative period, one to aorto-enteric fistula and another to septicemia. Other complications included stress-induced duodenal perforation (n=1) and endograft limb thrombosis requiring balloon angioplasty with Fogarty thrombectomy (n=1). Peri-procedural and long-term complications are depicted in Table 3.

**Table 3:** Peri-procedural and follow-up complications in both groups.

**Complications**	**TEVAR (n=61)**	**EVAR (n=40)**
Median follow-up, months (IQR)	36 (16-72)	48 (24-66)
Peri-procedural endoleak, *n* (%)	10 (16.3)	7 (17.5)
Equalizer balloon, *n* (%)	4 (6.5)	4 (10)
Vascular plugs, *n* (%)	1 (1.6)	1 (2.5)
Stent graft, *n* (%)	3(4.9)	0 (0)
Coiling, *n* (%)	0 (0)	2 (5)
Conservatively managed, *n* (%)	2 (3.2)	0(0)
Major perioperative complication, *n* (%)	11 (18.0%)	05 (12.5%)
Retrograde aortic dissection	01	--
Stent graft migration	02	--
Paraparesis	01	--
Sepsis	01	01
Device delivery failure	01	--
Lower Limb ischemia	02	01
Distal new entry tear	01	--
Aorto-enteric fistula	01	01
Acute renal failure	01	01
Duodenal perforation	--	01
30-day mortality *n* (%)	06 (9.8%)	02 (5%)
Follow-up complications		
Stent graft infection	01	01
Recurrence of Aorto-enteric fistula	01	---
Limb ischemia	01	---
Suspected aortic rupture	01	02
New onset endoleak	04	01
1-year mortality	12 (19.6%)	6 (15%)
Repeat interventions	03	0
Surgical	01	
Repeat endograft procedure	01	
Device closure	01	
Mean duration for repeat interventions after index procedure (months)	5.6 ± 1.73	

The most common intraoperative complication in the study was endoleak, observed in 17 (16.8%) patients. Most were type 1 endoleaks, successfully managed by balloon dilatation (n=8, 7.9%) and additional endograft stent deployment (n=3, 2.9%). The median postoperative follow-up was 36 months (interquartile range 16-72 months) and 48 months (interquartile range 24-66 months), in the TEVAR and EVAR groups respectively. Late complications included new-onset endoleak due to aortic enlargement in three patients, requiring repeat interventions. Late-onset type 1 endoleak was observed in one EVAR patient at nine years of follow-up, which was managed conservatively.

**Figure 2:**
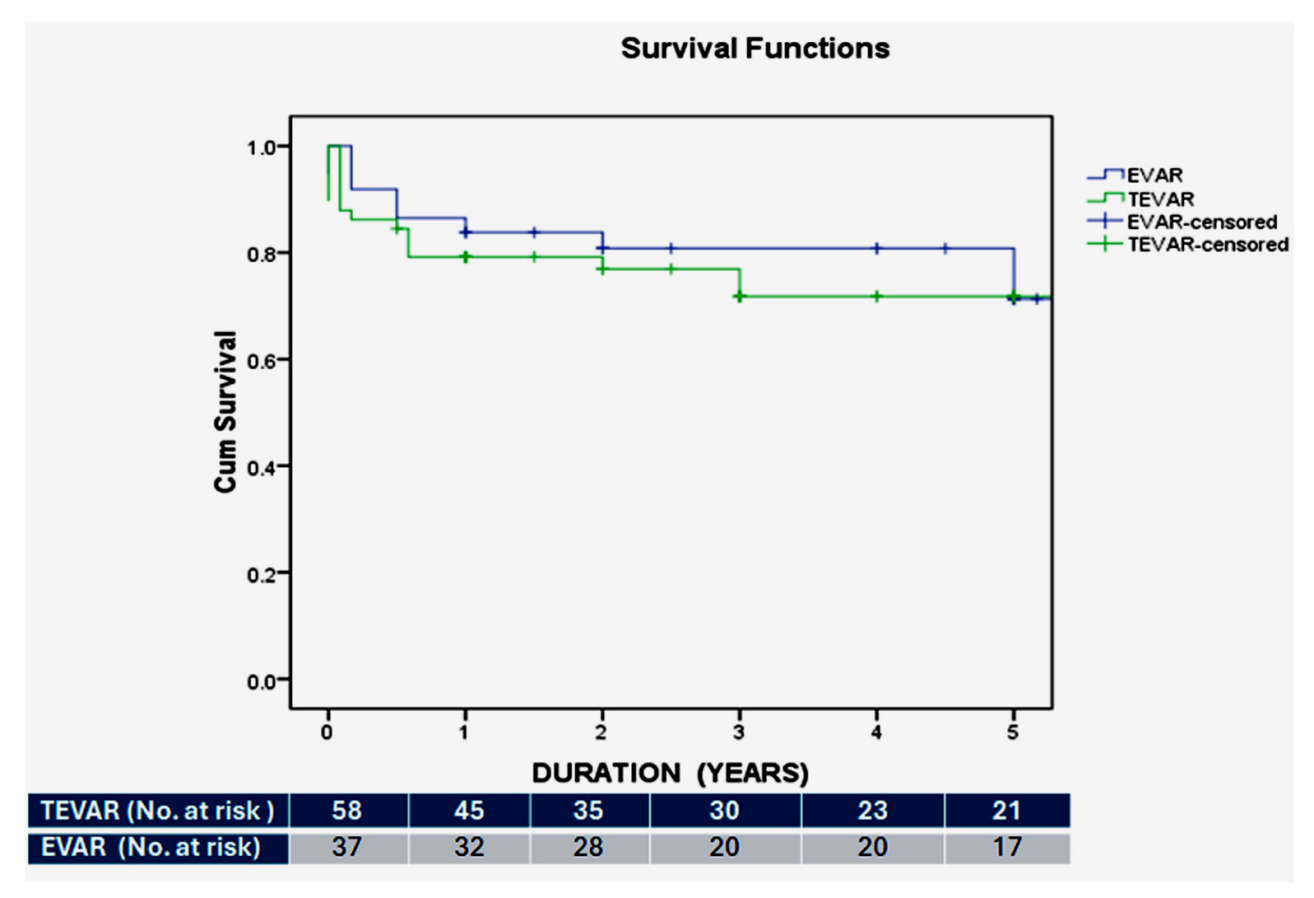
Kaplan–Meier survival curve for TEVAR and EVAR groups over 5 years. The difference was insignificant by the log-rank test, p=0.936.

**Figure 3:**
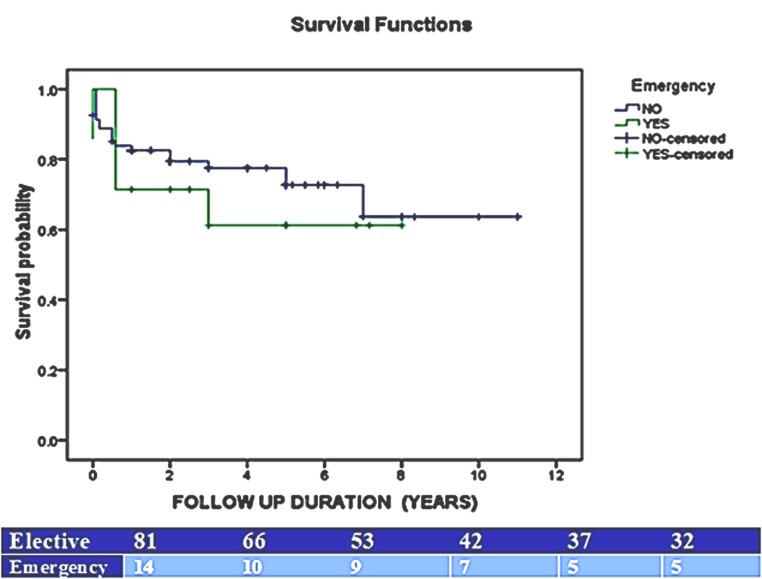
Kaplan–Meier survival curve for elective vs emergency cases of (T)EVAR over 5 years. The difference was insignificant by the log-rank test, p=0.443.

One-year and 5-year survival rates were 79% (95%CI 66.0-87.0, SE 0.053%) and 71% (95%CI 56.0- 81.0, SE 0.065%), respectively, for TEVAR patients and 84% (95%CI 67.0-92.0, SE 0.061%) and 69% (95%CI 46.0-83.0, SE 0.094%) for EVAR patients. Of the 101 patients in the cohort, 6 (5.9%) were lost to follow-up. In accordance with the intention-to-treat principle, all 101 patients were included in the primary survival analysis, with patients lost to follow-up censored at their last known contact. Figure 2 shows Kaplan-Meyer estimate curves depicting the survival functions for the two groups, with no significant difference (p=0.936). Multivariate Cox regression analysis regarding the predictors of long-term mortality demonstrated that diabetes and smoking were associated with worse outcomes in EVAR (HR 6.37; 95%CI 1.064-36.54; p=0.018) and TEVAR patients (HR 8.544; CI 1.048-49.644; p=0.045). A sensitivity analysis confirmed that exclusion of patients did not alter the significance of primary findings. The Kaplan-Meyer estimate curves depicting the survival between emergency vs. elective cases revealed nonsignificant differences (p=0.443), as shown in Figure 3.

## DISCUSSION

(T)EVAR offers an effective therapeutic option for several aortic pathologies, especially in high-risk surgical candidates.^14^ Studies comparing endovascular and surgical aortic repair have established comparable long-term outcomes, but some have expressed concerns over losing the survival advantage.^7,8^ Our study reassures that (T)EVAR offers safe and effective long-term outcomes for aortic pathologies with low rates of repeat interventions. We observed a similar long-term survival rate between TEVAR and EVAR groups, with TEVAR patients having an increased risk of peri-procedural complications. Other studies have also highlighted fundamental differences between the two procedures, with TEVAR associated with a younger population, higher postoperative mortality, morbidity, and more extended hospital stays.^15^ In our study, the mean age of the TEVAR group was younger than that of the EVAR group (52.8±15.2 vs. 66±12.9 years, p=0.001).

Hypertension, smoking, diabetes, and coronary artery disease were the most common risk factors identified in the study. Diabetes was associated with worse outcomes in patients with EVAR (HR 3.83;95% CI, 1.1-13.3; p=0.034). Diabetes-associated micro and macrovascular complications contribute to adverse consequences, including all-cause mortality.^16^ The other risk factors, such as emergency aortic repair, were not associated with increased periprocedural or late complications in the present study, but this could be due to a low number of enrolled cases. The presence of acute renal injury, stroke, anemia and advanced age are a few of the other risk factors associated with poor long-term outcomes,^17^ but none of these were statistically significant in the present study.

TEVAR is associated with higher rates of perioperative complications, including retrograde type A aortic dissection, distal aortic tear, stroke, visceral malperfusion, and access site complications due to large-bore delivery catheters.^18^ In a systematic review assessing the short-term complications of TEVAR, retrograde type A dissection was the most commonly observed graft-related complication, occurring in 3.1% of the cases.^19^ Grafts deployed in the aortic arch or more proximal Ishimaru zones (Zones 0-2) tend to spring back to their original shape, with consequences of endograft migration and tears at proximal or distal ends. We had one patient with proximal type A aortic dissection and another with distal aortic tear in the TEVAR group; both died due to complications. In a systematic review, Chen et al.^20^ reported a 2.5% incidence of retrograde type A aortic dissection, carrying a high mortality. The relative risk was highest in those with acute aortic dissection and use of a stent graft with proximal, extended bare stent struts.

Fernandez et al. reported a higher incidence of iliac artery rupture and avulsion in female patients with smaller caliber arteries.^21^ Although we conduct careful pre-procedure CT assessment of access vessel size, tortuosity, and calcification, one of the female TEVAR patients had delivery catheter entrapment at the right external iliac artery site, which was surgically removed. Endograft factors like device kinking and hostile iliac-femoral anatomy predispose to stenosis or thrombus formation in iliac stent graft limbs.^22^ One EVAR patient in our study had acute graft limb thrombosis on day 3, successfully managed with Fogarty thrombectomy. Another patient developed iliac artery occlusion at the site of insertion of a retro-peritoneal iliac conduit used to deliver a TEVAR device, which was managed with balloon angioplasty and stenting.^10^ Forceful introduction of oversized endovascular devices across compromised ilio-femoral arteries can lead to complications such as arterial rupture, avulsion, hematoma, and retroperitoneal bleeding.^10^ Use of conduits or bypass grafts in such hostile vascular access can prevent these and similar devastating complications. We used conduits or bypass grafts in eight patients.^10^

Patients with ruptured thoracic aorta pseudoaneurysm with aorto-esophageal or aortobronchial fistula are high-risk cases, presenting with massive hematemesis and rapid exsanguination. In a systematic review of TEVAR for aortic-esophageal fistula, Canaud et al. observed 30-day mortality of 19.4% and a 15% rate of endograft stent infection.^23^ We had two cases - one succumbed to in-hospital mortality and the other had endograft stent infection with recurrence of enteric fistula and succumbed to illness at 12 months of follow-up.^11^ We had three TEVAR patients with aorto-bronchial fistula presenting with massive hemoptysis. Two of them had a favorable long-term follow-up, while one died due to fulminant tuberculosis at five months of follow-up.^12^ In a systematic review of 134 aortobronchial fistula patients who underwent TEVAR, Canaud et al.^24^ reported 30-day mortality of 5.9%, with 17-month aortic and all-cause mortality of 14.3% and 21.4%, respectively. In a case series of 26 aortobronchial fistula patients, Kawaharada et al. reported 30-day mortality of 15%.^25^

Three TEVAR patients underwent aortic graft reinterventions at 5.6 ± 1.73 months of mean follow-up. Our study’s reintervention rate was low (2.9%) compared to the 11-32% rate reported in other studies.^26-28^ This could be due to the exclusion of complex cases of juxtarenal disease and those requiring fenestrated or branched grafts or in-situ fenestration. The five-year survival rate for EVAR patients ranges from 68%-73%,^29^ while for TEVAR, it ranges from 60-87%.^26-28^ We observed comparable 5-year survival rates of 69% in the EVAR group and 71% in the TEVAR group.

Certain limitations of the study exist. It is a single-center retrospective analysis, which may bias the results. We included both TEVAR and EVAR patients in the analysis because there were a limited number of patients, and these two treatments had different long-term outcomes. A post hoc sample size calculation indicated that approximately 350 and 296 patients would be required to estimate 5-year survival rates of 65%^17^ and 73%,^29^ for TEVAR and EVAR respectively, with a 5% margin of error at 95% confidence. Therefore, the present study is underpowered for precise survival estimation and to identify clinically meaningful associations in multivariate analysis. Patients with complex aortic diseases involving ascending aorta or abdominal aorta with visceral arteries requiring fenestrated or branched grafts were excluded from the study. Various confounding variables affect the long-term outcomes following the intervention; however, we were able to analyze limited risk factors in a small number of enrolled patients, as depicted in Table 4. In conclusion, we demonstrated that (T)EVAR is an effective and safe strategy with favorable long-term outcomes for selected aortic diseases in real-world practice.

**Table 4:** Multivariate model for all-cause mortality in the two groups.

**Variable**	**TEVAR**		**EVAR**	
	**HR (95% CI)**	**p-value**	**HR (95%CI)**	**p-value**
Hypertension	0.125 (0.015-1.051)	0.056	0.184 (0.010-3.531)	0.261
Diabetes	0.614 (0.059- 6.411)	0.683	6.37(1.064-36.54)	0.018
Male Sex	0.316 (0.030-3.315)	0.336	4.129 (0.36-32.0)	0.283
Coronary artery disease	2.139 (0.235-19.475)	0.500	0.903 (0.190-6.94)	0.922
Smoking	8.544 (1.048-49.644)	0.045	4.738 (0.429-51.357)	0.201
Chronic kidney disease	0.420 (0.109-2.33)	0.090	0.463 (0.012-1.526)	0.085
Peripheral vascular disease	1.595(0.812- 13.9)	0.673	1.92 (0.183-13.963)	0.566
